# Calcium oxalate crystal deposition in the kidney: identification, causes and consequences

**DOI:** 10.1007/s00240-020-01202-w

**Published:** 2020-07-27

**Authors:** R. Geraghty, K. Wood, J. A. Sayer

**Affiliations:** 1 Renal Services, The Newcastle Hospitals NHS Foundation Trust, Newcastle upon Tyne, NE7 7DN UK; 2 Histopathology Department, The Newcastle Hospitals NHS Foundation Trust, Newcastle upon Tyne, NE1 4LP UK; 3 Translational and Clinical Research Institute, Faculty of Medical Sciences, International Centre for Life, Newcastle University, Central Parkway, Newcastle upon Tyne, NE1 3BZ UK; 4 NIHR Newcastle Biomedical Research Centre, Newcastle upon Tyne, UK

**Keywords:** Calcium oxalate, Oxalosis, Primary hyperoxaluria, Enteric hyperoxaluria

## Abstract

Calcium oxalate (CaOx) crystal deposition within the tubules is often a perplexing finding on renal biopsy of both native and transplanted kidneys. Understanding the underlying causes may help diagnosis and future management. The most frequent cause of CaOx crystal deposition within the kidney is hyperoxaluria. When this is seen in native kidney biopsy, primary hyperoxaluria must be considered and investigated further with biochemical and genetic tests. Secondary hyperoxaluria, for example due to enteric hyperoxaluria following bariatric surgery, ingested ethylene glycol or vitamin C overdose may also cause CaOx deposition in native kidneys. CaOx deposition is a frequent finding in renal transplant biopsy, often as a consequence of acute tubular necrosis and is associated with poorer long-term graft outcomes. CaOx crystal deposition in the renal transplant may also be secondary to any of the causes associated with this phenotype in the native kidney. The pathophysiology underlying CaOx deposition is complex but this histological phenotype may indicate serious underlying pathology and should always warrant further investigation.

## Introduction

Calcium oxalate (CaOx) crystal deposition within the nephron [1–3], tubular cells [4] or interstitium [5] are sometimes found by the histopathologist examining a renal biopsy. CaOx, along with calcium phosphate (CaP) deposition may lead to nephrocalcinosis [6, 7], although in practice CaOx crystal deposition is often referred to as renal oxalosis or oxalate nephropathy. Bagnasco et al. examined biopsies of both native and transplanted kidneys over the course of 6 years [6]. Overall, 1% of native kidney biopsies and 4% of transplanted kidney biopsies demonstrated CaOx crystal deposition.

The presence of CaOx crystal deposition within a renal biopsy may indicate serious underlying pathology and indicate an underlying diagnosis that may not have previously been considered [7, 8]. Of particular relevance are the primary hyperoxalurias (PH), which may cause end stage kidney disease and may recur following kidney transplantation. The diagnosis of PH has potentially life-changing effects with a broad range of treatment options, up to and including dual kidney and liver transplant [9, 10].

Crystalluria, although associated with hyperoxaluria [11], is an uncommon finding [12–14]. There are no descriptions of the association between CaOx crystalluria and renal oxalosis. Here we aim to explore the causes of CaOx crystal deposition within a renal biopsy and therefore the implications and future management for the patient. We will review the histological appearances, the substrates that are most likely to cause CaOx crystal deposition and the pathophysiology associated with CaOx crystal deposition.

### Histology of calcium oxalate deposition

**Fig. 1:**
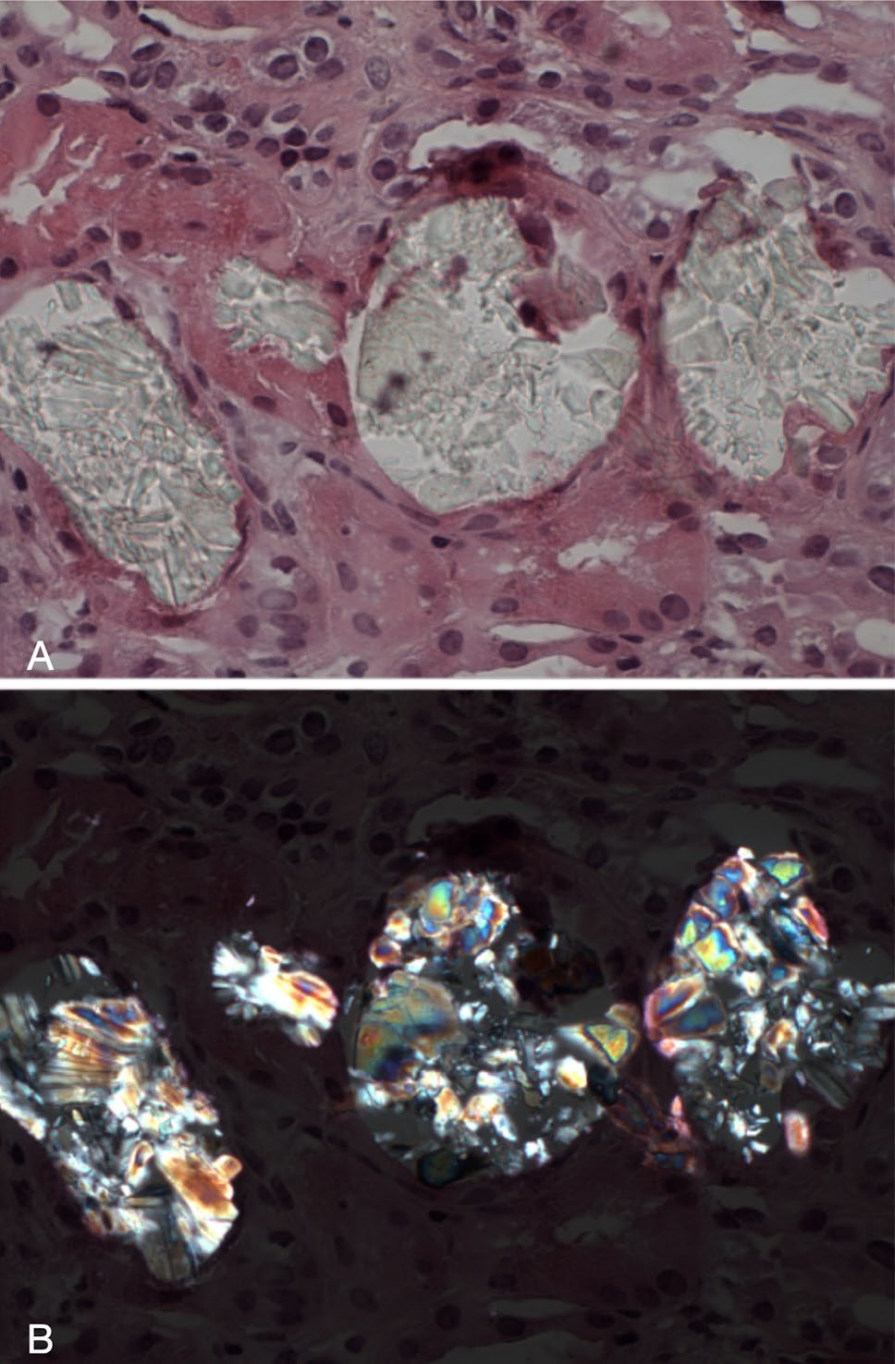
**a** = Oxalate nephropathy. A transplant kidney biopsy showing calcium oxalate crystals in dilated tubules. The crystals are clear with a refractile quality on routine microscopy (haematoxylin and eosin × 400). **b** = Oxalate nephropathy. The same calcium oxalate crystals exhibit bright birefringence when viewed under polarised light (polarised haematoxylin and eosin × 400)

Oxalate crystals precipitate in renal tubules causing tubular injury and in the longer term, interstitial fibrosis and tubular atrophy. They have a clear appearance on light microscopy [15] (Fig. 1a) but are much more easily seen when viewed under polarised light where they show bright birefringence (Fig. 1b). Particularly abundant crystals are typically associated with PH or ethylene glycol ingestion. Lesser degrees of deposition can be seen in a wide variety of conditions, which are discussed below. The main pathological differential diagnosis is 2,8 dihydroxyadenine crystalline nephropathy other cause of polarisable crystals seen in the kidney by the histopathologist. These patients, with biallelic mutations in *APRT*, have adenine phosphoribosyltransferase deficiency and often develop recurrent nephrolithiasis. Diagnosis can be challenging but the crystals can be distinguished from calcium oxalate crystals by their brown colour on haematoxylin and eosin staining [16].

### Calcium and oxalate: a tale of two substrates

Hypercalciuria and hyperoxaluria are both known to cause crystal deposition within the kidney [17]. In patients with hypercalciuria, the primary crystal deposited is CaP [2], this nidus may form the focus of aggregation for either CaP or CaOx [18] This variable aggregation has been demonstrated in vitro [19], in rat models [17, 20], and observed in humans [2]. However, in patients with hyperoxaluria the predominant crystal type is CaOx [21]; this has again been demonstrated in a rat model [17], in vitro [4, 5, 22] and in humans [2].

Crystal type and the components of subsequent aggregation are dependent upon specific locations along the nephron and degrees of supersaturation. In the urinary space, it seems that a CaP nidus initiates subsequent CaOx aggregation in the in vitro model [19], as in nephrolithiasis.

In the kidney, the type of crystal deposition appears to be different dependent on the location along the nephron. CaP crystals have been observed in the interstitium surrounding the ascending thin limb of the loop of Henle [2], in stone-forming patients with hypercalciuria. CaOx crystal deposition is typically seen more distally, having been observed within the collecting duct and the interstitium surrounding it [1, 14].

The situation therefore appears that in hypercalciuria, CaP crystals are deposited within and around the nephron, especially near the loop of Henle. By contrast, in hyperoxaluria, CaOx crystal deposition is found within collecting duct nephron segments. To test this hypothesis, Khan and Glenton examined hypercalciuric mice with increasing levels of oxaluria [20]. They demonstrated that in the genetic hypercalciuric stone-forming (GHS) rat model before dietary manipulation, only CaP crystals were formed. However, as the oxalate precursor hydroxyproline was added to their diet, CaOx crystals were observed. As hydroxyproline concentrations increased, inducing a hyperoxaluria, the crystal type switched to become entirely CaOx. This suggests that intrarenal CaOx crystal formation is dependent upon hyperoxaluria rather than hypercalciuria.

The mechanism of CaOx deposition within the kidney is subject to several factors. These include supersaturation and precipitation, crystal aggregation and deposition within the tubule/epithelium/interstitium. Several studies have demonstrated that hyperoxaluria induces intratubular precipitation of CaOx crystals located in the collecting duct [1, 23]. There are two potential mechanisms by which crystal passage through the tubule is inhibited (crystal retention). They have either aggregated and become too large [24, 25], or they have adhered to the epithelium [26]. Following either of these mechanisms, CaOx crystals then migrate into the epithelium [27] and interstitium [5]. The process behind this migration is unclear. However, crystal containing macrophages have been observed in both animal [28, 29] and human [30] epithelium/interstitium. Therefore active removal by macrophages is a possible mechanism for this observation, although this has yet to be demonstrated.

### Pathologies associated with calcium oxalate crystal deposition

CaOx crystal deposition may be noted in both native and transplanted kidneys, as a consequence of hyperoxaluria. Oxalate has both endogenous and exogenous sources [31, 32] and both are equally able to induce hyperoxaluria (defined as > 40–45 mg per 24 h or > 0.45–0.5 mmol per 24 h). Tubular CaOx deposition leading to acute or chronic tubular injury, interstitial fibrosis and progressive renal insufficiency is termed oxalate nephropathy or renal oxalosis.

Both native and transplanted kidneys are susceptible to hyperoxaluria and subsequent oxalate nephropathy and the causes for hyperoxaluria and crystal deposition differ (Table 1).

**Table 1:** Causes of Calcium Oxalate crystal deposition within the native and transplanted kidney

Calcium oxalate crystal deposition	
**Native kidney**	**Transplanted kidney**
Primary hyperoxaluria – types 1–3	Causes as per native kidney
Secondary hyperoxaluria:	Transient hyperoxaluria due to sudden increase in GFR and previous systemic oxalosis secondary to end stage kidney disease
Enteric hyperoxaluria (fat malabsorption)	Acute tubular necrosis
High oxalate diet	Chronic allograft nephropathy
Ethylene glycol intoxication	
Thiamine/Pyridoxine deficiency	
Vitamin C overdose (precursor of oxalic acid)	
Orlistat use	
Alterations in intestinal flora	
Genetic variations of oxalate transporters	
Acute tubular necrosis	

On light microscopy 2,8-hydroxyadenine crystals may mimic CaOx crystals under polarized light, because of their high birefringence [15]. However, the finding of 2,8-hydroxyadenine crystals mimicking CaOx crystals can lead to a rare, often missed and important genetic diagnosis being made. Likewise, genuine CaOx deposition can lead to other important diagnoses being made and should never be ignored.

Diabetes mellitus is a common cause of nephropathy and it is unclear whether it is associated with renal oxalosis. Diabetics have demonstrably higher urinary oxalate concentrations than healthy patients [33]. They have also been observed to develop oxalate nephropathy in several case reports [34, 35]. However, in these case reports, the patients had independent risk factors for renal oxalosis including Roux-en-Y bypass and increased dietary oxalate. Moreover, CaOx crystals are not among the number of histological features of diabetic nephropathy [36, 37]. A large study of cadaveric renal biopsies examined risk factors associated with renal oxalosis [38] Diabetes mellitus was shown not to be associated with renal oxalosis. Therefore, if CaOx crystals are seen on renal biopsy of a patient with diabetes, the likely driving factor is hyperoxaluria. The type and cause of hyperoxaluria should therefore be investigated as this may lead to important changes in patient management.

### Primary hyperoxaluria

Primary hyperoxaluria is a rare autosomal recessive disorder associated with renal CaOx crystal deposition. Oxalate is an end metabolite for glyoxylate and the three types of primary hyperoxalurias (PH1-3) affect enzymes of glyoxylate metabolism. The enzymes implicated are: alanine glyoxylate aminotransferase (PH1) [39], glycolate reductase/hydroxypyruvate reductase (PH2) [40] and 4-hydroxy-2-ketoglutarate aldolase (PH3) [41, 42]. These disorders tend to present in childhood to early adolescence with severe recurrent nephrolithiasis, although given some may be asymptomatic (especially PH3), they may not present until the development of advanced renal failure. PH may also present in late adult life with calcium oxalate stone formation or insidious chronic kidney disease.

The majority of cases are PH1, which have the most severe disease phenotypes. PH1 and PH2 both cause progressive nephrocalcinosis, nephrolithiasis and renal damage resulting in early end stage renal failure [13, 26–28]. With the progressive decline in renal function comes rising plasma oxalate levels. At a glomerular filtration rate < 45 ml/min/1.73 m^2^ plasma oxalate concentrations exceed the supersaturation threshold leading to systemic deposition of CaOx (systemic oxalosis) [43]. This leads to early death if left untreated [44].

It is unclear if patients with PH3 have the same natural history as PH1/2 given its rarity and recent description. Recent data has shown children with PH3 show a decline in renal function [45]. However, there remains a lack of long-term follow-up data to allow for an accurate description of its clinical course. It is possible that all types of PH may present with unexplained chronic kidney disease and CaOx crystal deposition on renal biopsy.

### Secondary hyperoxalurias

Secondary hyperoxaluria may be due to a number of different causes. The passage of oxalate through the body helps illustrate why differing mechanisms cause hyperoxaluria. There is a large oxalate content in certain foods [46], which is both metabolized by gut commensals (*Oxalobacter formigenes*) [47] and absorbed into the enterohepatic circulation [48, 49]. Absorbed oxalate is then filtered and excreted in the kidney [48, 49] along with oxalate produced as an end-point of glyoxylate metabolism.

At each of these points, excess oxalate may occur. Case reports describing high intakes of oxalate containing foods [46] or vitamin C [50] (which is catabolized into oxalate) are associated with hyperoxaluria. Deficiencies, dietary or otherwise, in thiamine or pyridoxine [51–54], deliberate ingestion of orlistat [55] or ethylene glycol [56, 57] may also lead to hyperoxaluria. High doses of vitamin C [50], some foods [58–61], excessive dieting [62] and ethylene glycol [56] have been demonstrated to induce acute oxalate nephropathy.

The gut commensal *Oxalobacter formigenes*, catabolizes oxalate thus diminishing gut absorption [63, 64]. There has been an attempt to exploit this phenomenon for PH, which showed initial promise, but unfortunately failed in phase II/III trials [65]. Although touted as a treatment, there have not been further studies of its effectiveness to treat secondary hyperoxaluria.

Several case reports have associated hyperoxaluria with bariatric surgery [66, 67] as well as chronic pancreatitis [68, 69], with both conditions associated with acute oxalate nephropathy [66, 68]. Increased oxalate absorption is a function of fat malabsorption (enteric hyperoxaluria). In the normal state, oxalate is bound to calcium within the gut. Fat malabsorption leads to free fatty acids binding to calcium, leaving the oxalate in its absorbable, ionised state [49].

Mice and humans with genetic variations of gut oxalate transporters have also been demonstrated to have increased urinary oxalate [70] Deletion of *Slc26a6* in mice [71, 72] along with variants V185M in the *SLC26A6* transporter in humans [73] have both been associated with hyperoxaluria. None of these studies performed renal biopsies and therefore further study is required to see if these are risk factors for oxalate nephropathy and CaOx deposition.

### Transplanted kidneys

Around 4% of transplanted kidneys will display CaOx crystals on biopsy [6]. Crystals can be found early or late, distributed throughout the kidney or only in focal segments.

In the initial post-operative period it is thought that, due to the poor renal function indicating the need for transplant, there is systemic oxalosis. With the improvement in renal function attained by transplantation there is rapid excretion of the excess oxalate. This leads to a transient hyperoxaluria with a small proportion developing subsequent renal precipitation of CaOx [74]. There is debate as to whether or not this initial transient hyperoxaluria is pathological, and long-term outcomes of this have not been proven.

There is more evidence for the implications of CaOx crystals on renal biopsy, albeit conflicting. In the short term, the presence of CaOx crystals on graft biopsy up to 3 months after transplantation seems to be associated with poorer longer term graft survival [75]. Although a later study demonstrated that, although graft function at 1 year was significantly poorer in those with CaOx deposition, there was no statistically significant difference in renal function at 2 years [6]. In this second study however, there was an overall drop in both control and crystal graft function in the second year compared to the first. It is likely that CaOx crystals are a negative prognostic indicator for long-term graft survival in the initial period following transplantation. These patients should be followed-up closely.

Delayed graft function and acute tubular necrosis (ATN) or acute cell‐mediated rejection is associated with focal CaOx deposition [76, 77]. The long-term impact of these acute events is unclear. The majority of transplanted kidneys demonstrated normal function at follow-up [76]. However, these observations were underpowered, lacked follow-up biopsies, and biochemical data for clinical correlation. The authors postulated this observation was due to high oxalate excretion using the mechanism previously described. However, inferring this mechanism from the data is difficult due to the lack of clinical context and small numbers of patients.

In the longer term, CaOx crystals are seen on biopsy of those with chronic allograft nephropathy [76]. In the two patients studied, CaOx crystal deposition was widespread in keeping with chronic renal failure (mechanism discussed below). An earlier study by Memeo et al. of forty allograft nephrectomies showed 87% had widespread CaOx crystals [78]. Again, given the low numbers it is difficult to draw conclusions from these case reports, but they suggest CaOx crystals, identified at any point in time from biopsy, are associated with long-term graft failure.

Transplanted kidneys can also be affected by any of the primary or secondary hyperoxalurias. Failure to diagnose PH prior to transplantation may result in early graft failure [79, 80]. Likewise for secondary hyperoxalurias, failure to recognise may lead to acute kidney injury [81] or even graft failure. There have been graft failure case reports for enteric hyperoxaluria [82, 83] and excessive vitamin C intake [84].

### Pathophysiology of renal damage associated with crystal deposition

Severe hyperoxaluria has been demonstrated to be clinically associated with acute or chronic renal failure, although it is unclear which is causative of the other. It is also unclear whether mild to moderate hyperoxaluria, such as that seen in PH3, is associated with renal damage, despite evidence of CaOx crystal deposition in both conditions.

There is a large body of evidence from rat and in vitro models, and human observation that CaOx crystal deposition is associated with renal epithelial damage [4, 5, 85–89]. Differing structures of CaOx crystals can damage renal epithelial cells inducing apoptosis [22]. This body of evidence suggests that epithelial injury and progressive inflammation is caused by CaOx crystals, rather than CaOx crystals forming secondary to renal damage. This explains the findings in PH and severe secondary hyperoxaluria.

The observation that CaOx crystals are only found in focal segments of acute tubular necrosis in transplanted kidneys [76, 77] however, does not fit with the widespread renal damage and CaOx crystals of hyperoxaluria. It implies that CaOx crystal deposition seen in this situation is secondary to focal epithelial damage [4], rather than crystal precipitation and subsequent epithelial damage.

The pathophysiology of renal oxalosis secondary to severe hyperoxaluria has been described. However, the mechanism of focal CaOx crystal deposition in acute tubular necrosis remains unclear. CaOx crystals on renal biopsy should always prompt investigation for serious underlying conditions in both the native and transplanted kidney (Table 1), that could lead to progressive renal failure.

## Conclusion

CaOx crystals identified histologically on renal biopsy are indicative of a potential underlying pathology. This finding warrants further investigation to determine the cause, the most serious of which is PH. Much of the clinical literature describing conditions associated with CaOx crystal deposition are case reports. In the long-term there appears to be a potential association between CaOx deposition and increased risk of chronic kidney disease. Larger studies are needed to examine this association in more depth.
